# Silymarin Constituent 2,3-Dehydrosilybin Triggers Reserpine-Sensitive Positive Inotropic Effect in Perfused Rat Heart

**DOI:** 10.1371/journal.pone.0139208

**Published:** 2015-09-29

**Authors:** Eva Gabrielová, Aleksey Vladimirovich Zholobenko, Lenka Bartošíková, Jiří Nečas, Martin Modriansky

**Affiliations:** 1 Department of Medical Chemistry and Biochemistry, Faculty of Medicine and Dentistry, Palacký University, Olomouc, Czech Republic; 2 Department of Physiology, Faculty of Medicine and Dentistry, Palacký University, Olomouc, Czech Republic; 3 Institute of Molecular and Translational Medicine, Faculty of Medicine and Dentistry, Palacký University, Olomouc, Czech Republic; Max-Delbrück Center for Molecular Medicine (MDC), GERMANY

## Abstract

2,3-dehydrosilybin (DHS) is a minor flavonolignan component of *Silybum marianum* seed extract known for its hepatoprotective activity. Recently we identified DHS as a potentially cardioprotective substance during hypoxia/reoxygenation in isolated neonatal rat cardiomyocytes. This is the first report of positive inotropic effect of DHS on perfused adult rat heart. When applied to perfused adult rat heart, DHS caused a dose-dependent inotropic effect resembling that of catecholamines. The effect was apparent with DHS concentration as low as 10 nM. Suspecting direct interaction with β-adrenergic receptors, we tested whether DHS can trigger β agonist-dependent gene transcription in a model cell line. While DHS alone was unable to trigger β agonist-dependent gene transcription, it enhanced the effect of isoproterenol, a known unspecific β agonist. Further tests confirmed that DHS could not induce cAMP accumulation in isolated neonatal rat cardiomyocytes even though high concentrations (≥ 10 μM) of DHS were capable of decreasing phosphodiesterase activity. Pre-treatment of rats with reserpine, an indole alkaloid which depletes catecholamines from peripheral sympathetic nerve endings, abolished the DHS inotropic effect in perfused hearts. Our data suggest that DHS causes the inotropic effect without acting as a β agonist. Hence we identify DHS as a novel inotropic agent.

## Introduction

2,3-dehydrosilybin (DHS) is a minor constituent of silymarin, a well studied extract from seeds of milk thistle (*Silybum marianum* L.). The extract, and especially its major constituent silybin, is considered a hepatoprotective agent [1,2]. Besides hepatoprotective activity, silymarin has been shown to protect against ischemia-reperfusioninduced myocardial infarction [3] and oxidative stress in rat cardiac tissues [4]. Silymarin is, however, a mixture of polyphenolic compounds each of which may be responsible for the cardioprotective activity of the whole extract [5]. Indeed, a study testing cardioprotection against doxorubicin cardiotoxicity concluded that individual silymarin constituents displayed better efficacy than the whole extract [6]

Silymarin extract demonstrated protective effects against acrolein- induced cardiotoxicity in mice [7] and doxorubicin [8], cis-platin [9], and adriamycin-induced cardiotoxicity in rats [10]. All of these studies indicated that oxidative stress or oxidative stress related parameters were attenuated by silymarin treatment. However, the positive effect may not be limited to direct interaction of the extract or its constituents with oxygen radicals. Therefore studies aimed at individual substances and their biological activities were performed.

Our recent research suggests that DHS may be responsible for some biological activities ascribed to the silymarin extract. We have shown that DHS displays uncoupler-like activity in isolated mitochondria [11] and attenuates reactive oxygen species formation during hypoxia/reoxygenation in primary neonatal rat cardiomyocytes [12]. The latter activity was associated with modulation of PKC*ε* phosphorylation, where DHS restored the phosphorylated/total PKC*ε* ratio to the levels observed in control cells. Because PKC*ε* is a major player in maintaining intramitochondrial signaling [13], we perceived this effect of DHS as solid basis for further investigation in whole hearts.

Heart contraction is regulated by inotropic substances or inotropes. Positive inotropes, i.e. catecholamines such as norepinephrine, stimulate heart muscle contraction and cause heart rate to increase. Clinically, positive inotropes are used under certain heart failure conditions where adrenergic drive for support of cardiac function is necessary, e.g. for hemodynamically unstable heart failure patients or patients displaying low cardiac output syndrome in congenital heart disease. Positive inotropes are unsuitable for chronic heart failure as sustained adrenergic stimulation will lead to arrhythmias, but may benefit patients with acute heart failure, especially with clinically evident hypoperfusion, cardiogenic shock or as a bridge to more definitive treatments [14].

Here we report for the first time that DHS causes positive inotropic effect in perfused adult rat heart. The effect appears to depend on endogenous catecholamines.

## Materials and Methods

### 2.1 Chemicals and test compounds

Dulbecco’s modified Eagle’s medium (DMEM), heat-inactivated fetal bovine serum (FBS), stabilized penicillin-streptomycin solution (PenStrep), sterile dimethylsulfoxide (DMSO), norepinephrine (NE), isoproterenol (ISO), propranolol (PRO) and reserpine (RES) were obtained from Sigma-Aldrich (St. Louis, MO, USA). 3-isobutyl-1-methylxanthine (IBMX) was supplied by Enzo Life Sciences (Farmingdale, NY, USA) as part of the phosphodiesterase assay kit.

2,3-dehydrosilybin (DHS, 98%; C_25_H_20_O_10_, Mr 480) was prepared and provided by prof. Vladimír Křen at the Institute of Microbiology, Academy of Science of the Czech Republic, Prague, Czech Republic. The method of DHS preparation is based on base-catalyzed disproportionation [15]. Brief summary of the preparation: silybin (2.5 g, 5.183 mmol) and NaHCO_3_ (1.74 g, 20.798 mmol) were dissolved in methanol (100 ml) and the mixture was heated under reflux for 16 h. The mixture was then left to cool to room temperature and poured into ice-cold water containing HCl (400 ml, 5% v/v). The precipitate formed was filtered off, washed with H_2_O, dissolved in a mixture of ethyl acetate/acetone (1:1), and evaporated to give 2.17 g of dry residue. The solid was crystallized from methanol (1000 mg, 40% yield). The mother liquor was filtered through a silica gel pad (CHCl_3_/acetone/HCOOH 90:10:1–70:30:1) to obtain, after concentration, another portion of the product, which after recrystallization from methanol yielded pure DHS (270 mg, 11%). Thus, the total yield of DHS was 51%. Stock solution of DHS (10 mM) was prepared in DMSO. The final concentration of DMSO in the medium was 0.5% (v/v).

### 2.2 Animals

Wistar rats (300 g) were bred and housed in a certified animal house in accordance with European Guidelines on Laboratory Animal Care and policy of the ethics committee of the Faculty of Medicine and Dentistry, Palacký University. Animal treatment and sacrifice procedure for neonatal rats was approved by the Ethical Committee for Laboratory Animal Treatment of the Faculty of Medicine and Dentistry, Palacký University. The study was approved by the Ethical Committee for Laboratory Animal Treatment of the Faculty of Medicine and Dentistry, Palacký University, under the title “Elucidation of the mechanism of flavonoid-dependent cardioprotection and changes in the bioenergetic status of the cell” and filed under number 10413/2010-30.

### 2.3 Perfusion protocol

The rats (male Wistar, 300 g, 10 weeks) were anesthetized with an i.p. injection of an anaesthetic mixture (2% Rometar 0.5 ml + 1% Narkamon 10 ml, dose 0.5 ml solution/100 g body weight). After the i.p. heparine injection of 500 IU dose, the hearts were excised and perfused. In all experiments, the modified Langendorff method and the universal apparatus Hugo Sachs Electronic UP 100 (Germany HSE) were used. Hearts were perfused with a modified Krebs-Henseleit solution containing 118 mM NaCl, 5.9 mM KCl, 1.75 mM CaCl_2_, 1.2 mM MgSO_4_, 0.5 mM EDTA, 25 mM NaHCO_3_, 16.7 mM glucose (pH 7.4). The perfusate was gassed with 95% O_2_ - 5% CO_2_, which resulted in a Po_2_ > 600 mmHg at the level of the aortic cannula and a buffer pH 7.4. Schedule: stabilization/compound perfusion/reperfusion proceeded at intervals of 10/15/10 min. Left ventricle pressure (LVP), left ventricle end diastolic pressure (LVEDP), and contractility (dP/dtmax) were measured using a ball filled with liquid (8–12 mmHg), inserted through the left atrium into the left ventricle and connected to an analog convertor Isotec HSE, DIF modul HSE (Harvard apparatus GmbH, March-Hugstetten, Germany) [16,17].

### 2.4. Reserpine treatment of animals

The effect of catecholamine-depleting pretreatments, reserpine (0.3 mg/kg i.p. 24 h before the experiments), on left ventricular pressure (LVP) and the inotropic response to DHS was studied in isolated perfused rat heart. Rats were divided into two groups (nonreserpinised/reserpinised). Hearts were excised following a standard anesthetic procedure, placed into ice-cold perfusion buffer and subsequently perfused according to Langendorff at 37°C with Krebs-Henseleit buffer (118 mM NaCl, 5.9 mM KCl, 1.75 mM CaCl_2_, 1.2 mM MgSO_4_, 0.5 mM EDTA, 25 mM NaHCO_3_, 16.7 mM glucose, pH 7.4). Each heart was allowed to stabilize for 10 min. After the stabilization period, the hearts were subjected to a specific protocol: A 15-min period of tested compound perfusion, followed by a period of 10 min reperfusion in all groups. Left ventricular developed pressure (LVDP) and contractility (dP/dt_max_) were measured. We tested DHS at concentrations of 10 nM, 100 nM, 1 μM and 10 μM.

### 2.5. Isolation of neonatal rat cardiomyocytes

The procedure described by Chlopcikova et al. [18] was followed. Entire hearts were excised from 2–5 day old rats that were subjected to CO_2_ treatment followed by decapitation. The hearts were immediately minced in a balanced salt solution containing: 20 mM HEPES, 120mM NaCl, 1 mM NaH_2_PO_4_, 5.5 mM glucose, 5.4 mM KCl and 0.8 mM MgSO_4_ (pH 7.3–7.4). Tissues were then digested in trypsin and the released cells resuspended in a medium containing Dulbecco’s Modified Eagle Medium (DMEM) and a medium 199 (4:1) supplemented with horse serum (10%), fetal calf serum (5%), penicillin (100 U/ml) and streptomycin (100 μg/ml). The suspension enriched in non-adhesive myocytes was transferred to collagen I-coated culture dishes at a density of 5×10^4^ cells per cm^2^. Cells were incubated in 95% air and 5% CO_2_ at 37°C. Culture medium was removed after 72 h and replaced with another containing DMEM and medium 199 (4:1) with penicillin (100 U/ml) and streptomycin (100 μg/ml). Cultured cardiomyocytes were allowed to reach a confluence before being used experimentally. The percentage of beating myocardial cells exceeded 85–90% after 3 days in culture for each experiment.

### 2.6. Cultivation and transfection of H9c2 cells

For CRELuc reporter assays undifferentiated H9c2 cells, which were not serum starved prior to treatment, were used. H9c2 cells were obtained from European collection of cell cultures (ECACC catalogue No 88092904; rat DB1X heart myoblast). Cells were grown in DMEM supplemented with 10% fetal calf serum (FCS), 1% Non-essential amino acids (NEAA), 50 μg/ml Penicillin-Streptomycin (PenStrep). Cells were passaged at 1:3 when confluence reached 80%. For CRE-Luciferase assays, cells between passages 5 and 20 were trypsinised and co-transfected with pCRELuc (2.2 μg per 10^6^ cells- firefly luciferase) and pCMVGal (0.2 μg per 10^6^ cells) while suspended in OptiMEM (Gibco Life Technologies, Prague, Czech republic) at 2×10^5^ cells per ml. pCRELuc and pCMVGal plasmids were obtained from PathDetect system (Stratagene, La Jolla, CA, USA). Cells were subsequently plated on 24 well plates at 10^5^ cells per well. Following 4 h incubation (37°C, 5% CO_2_) cells were rinsed with PBS and incubated in complete medium (DMEM + 50 μg/ml PenStrep + NEAA + 10% FCS) for 24 h. Medium was then exchanged for an equal volume of complete medium with appropriate concentrations of treatment drugs. 1 μM ISO, 100 μM NE and various concentrations of DHS were applied alone or in the presence of PRO. 1 μM DHS was also combined with 1 μM ISO to treat the cells in the presence and in the absence of PRO. Cells were incubated (37°C, 5% CO_2_) for a further 24 h (in complete medium with treatments), rinsed twice in PBS and lysed in lysis buffer (100 mM Potassium Phosphate, 0.1% Triton-X 100, pH 7.8), prior to luminometry by Dual Light system (Applied Biosystems Life Technologies, Prague, Czech republic).

### 2.7. Phosphodiesterase inhibition assay

To test for possible inhibition of phosphodiesterase we used the BIOMOL Cyclic Nucleotide Phosphodiesterase (PDE) assay kit (Enzo Life Sciences, Farmingdale, NY, USA). We followed the manufacturer's protocol for testing inhibitors of PDE, where the incubation temperature was 30°C and incubation time was 30 min. IBMX, a general inhibitor of PDEs, was used at 50 μM as a positive control of inhibition. We also used the manufacturer's protocol to confirm that DHS did not interfere with the release of phosphate by 5´-nucleotidase at any of the concentrations tested. The absorbance developed following BIOMOL green application was measured at 620 nm with a plate reader (InfititeM200pro, Tecan Instruments, Mannedorf, Switzerland).

### 2.8. cAMP accumulation assay

Neonatal rat myocytes were plated on 24 well plates at the density of 5×10^4^ cells per cm^2^ in myocyte growth medium. Growth medium was aspirated from wells and cells were incubated in fresh medium containing flavonoids and control agonists for 5 min. Cells were then rinsed with ice cold PBS and lysed by 20 min incubation in 0.1 M HCl. Cell lysates were spun at 1000*g* for 10 min. Supernatants were then used for cAMP detection using a cAMP EIA kit (Cayman chemicals), as per the manufacturer's recommended protocol. In brief, supernatants were diluted 3:7 in assay buffer (62 mM phosphate, 400 mM NaCl, 1.5 mM NaN_3_, 1mg/ml BSA) and incubated on EIA plates at 4°C overnight with tracer and cAMP antiserum. The plate was then rinsed 5× with wash buffer (6.2 mM phosphate, 0.05% polysorbate 20) and detection was performed using Ellman's method, with absorbance change subsequently measured at 420 nm on a plate reader (InfiniteM200pro, Tecan Instruments, Mannedorf, Switzerland).

### 2.9. Statistical analysis

2-Way analysis of variance (unbalanced design) with post-hoc Tukey test was applied using STATISTICA software (StatSoft, Tulsa, OK, USA). p<0.05 was considered statistically significant.

## Results

### 3.1. DHS causes positive inotropic effect in perfused rat hearts

**Fig 1:**
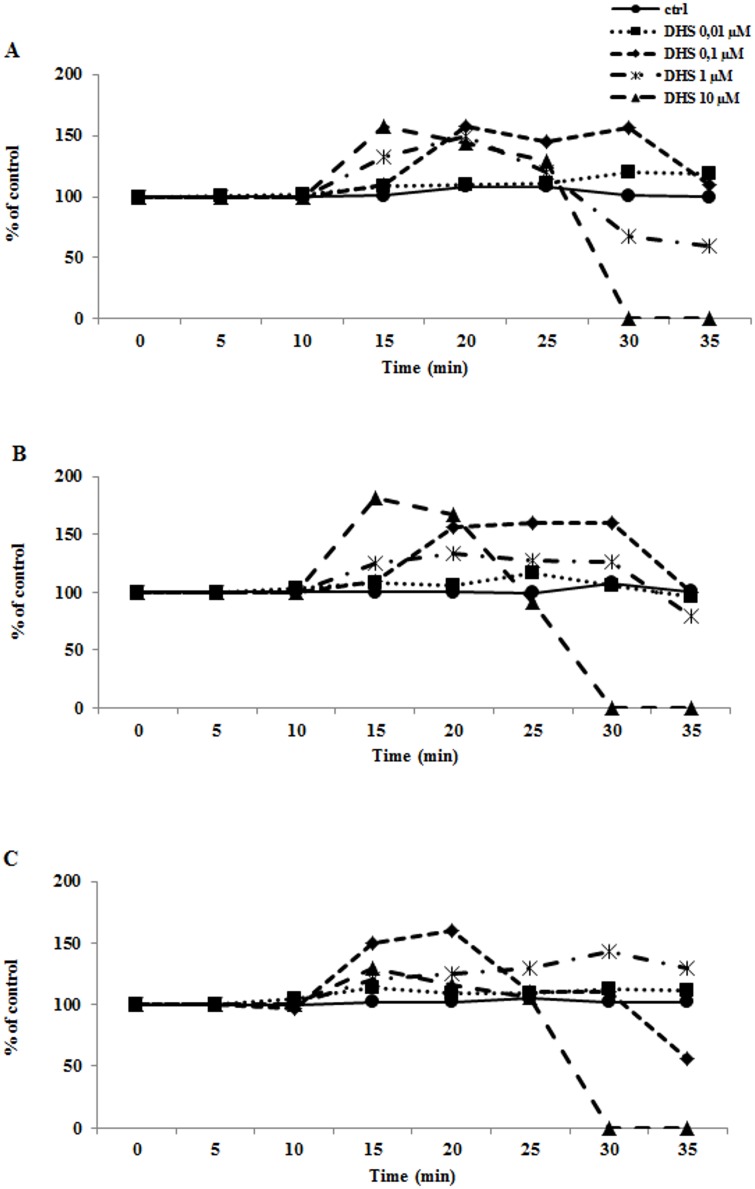
Effect of DHS on biomechanical parameters in perfused adult rat heart. Isolated adult rat hearts were perfused with Krebs-Henseleit buffer for 10 min followed by 15 min perfusion with DHS or Krebs-Henseleit buffer in control hearts, and concluded by 10 min perfusion (wash-out) with Krebs-Henseleit buffer. Data presented were obtained for control hearts (●, full line) and hearts treated with: 10 nM DHS (■, dotted line); 100 nM DHS (◆, dashed line); 1 μM DHS (*, dash-and-dot line) and 10 μM DHS (▲, dashed line). Individual panels are: A—LVP; B—contractility; C—heart rate. Each point represents an average for three experiments.

Langendorff perfused hearts are the model of choice for testing the effect of a substance on the whole organ. Basing the initial concentration on our previous work in isolated cardiomyocytes [11,12], we used this perfusion model to observe the effect of 10 μM DHS, on the whole organ as a prerequisite for cardioprotective work. To our surprise, addition of 10 μM DHS to the perfusion solution caused an immediate increase in blood pressure, contractility, and heart rate (Fig 1). A dramatic morphological change, apparent as local swelling and amorphous bulges, was observed in the heart following 10 min of perfusion with 10 μM DHS, accompanied by arrhythmias, fibrillation, and eventually cardiac arrest, which is shown as 0% activity on all three panels of Fig 1. The effect was not reversed upon washout of DHS and resembled profound, sustained adrenergic stimulation that results in exhaustion of the organ, sometimes called “oxygen wasting”. Also, rapidity and extent of the DHS effect suggested stimulation of β-adrenergic receptors and/or interaction with β-adrenergic pathway. The inotropic effect was less pronounced at lower concentrations of DHS, where, following the 15 min of substance perfusion, biomechanical parameters gradually returned to values observed in control hearts. The lowest DHS concentration tested, i.e. 10 nM, did not affect contractility throughout the perfusion but retained the increased LVP and heart rate even during washout.

**Fig 2:**
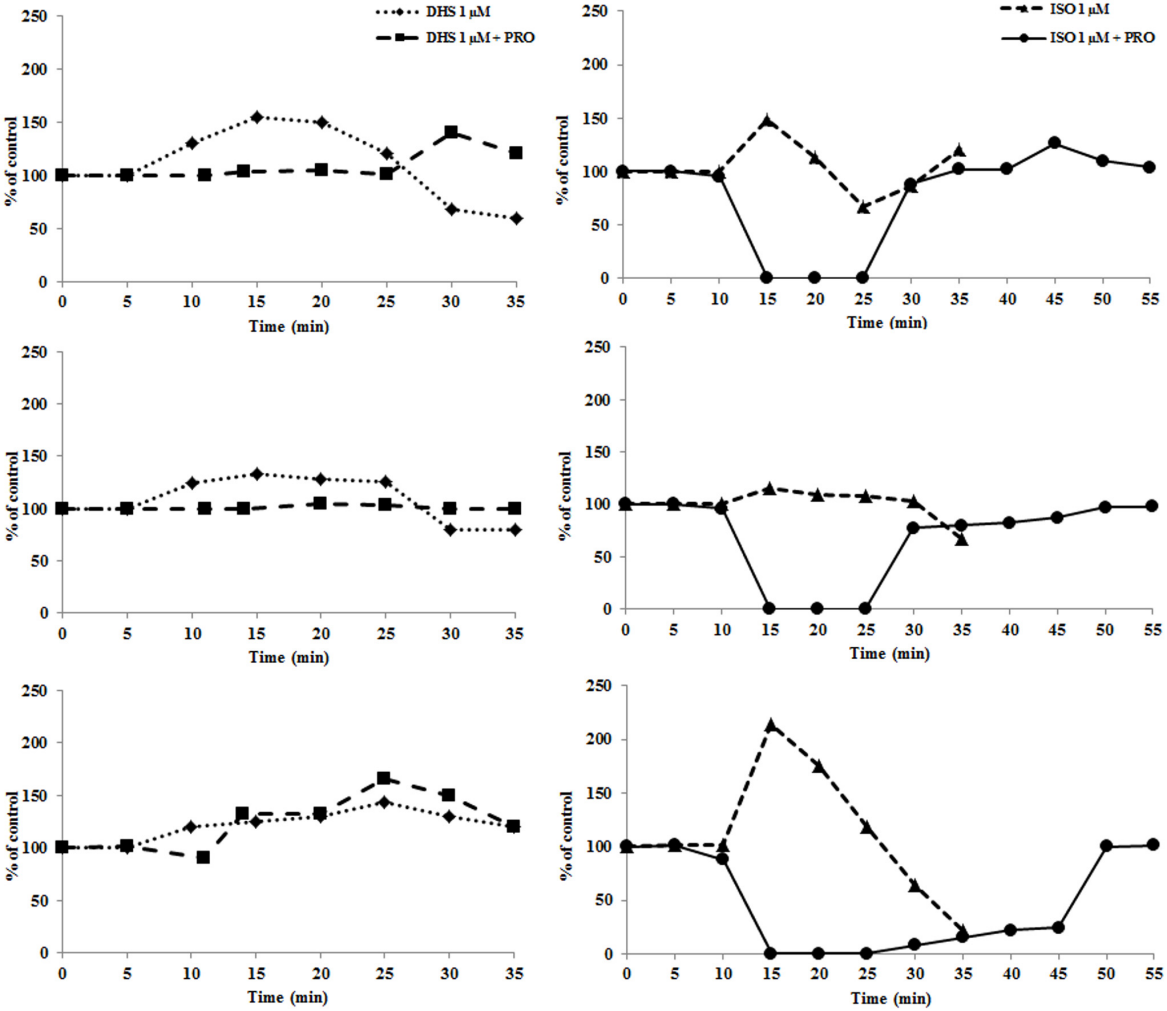
Effect of propranolol on perfusion treatments. Isolated adult rat hearts were perfused with Krebs-Henseleit buffer for 10 min followed by 15 min perfusion with DHS or DHS + PRO and concluded by 10 min perfusion (wash-out) with Krebs-Henseleit buffer. Data presented were obtained for hearts treated with 1 μM DHS (◆, dotted line) and 200 μM PRO together with 1 μM DHS (■, dashed line). Individual panels are: A—LVP; B—contractility; C–heart rate. The same perfusion protocol was used for ISO treatment. Data presented were obtained for hearts treated with 1 μM ISO (▲, dashed line) and 200 μM PRO together with 1 μM ISO (●, full line). Individual panels are: D—LVP; E—contractility; F—heart rate.

The swiftness of the effect and resemblance to catecholamine stimulation led us to investigate whether DHS effect may be modulated by β-antagonist. Because propranolol is a known, non-selective β-antagonist we tested the effect of DHS in its presence. 200 μM propranolol (PRO) was able to diminish the effect of 1 μM DHS in LVP and contractility parameters, without attenuating the effect on heart rate (Fig 2). Isoproterenol (ISO), a known β-agonist, was used for comparison because it triggered a profound inotropic effect in perfused hearts at 1 μM concentration. This effect was attenuated by 200 μM PRO as with DHS.

All the data suggested a possible interaction of DHS with β-adrenergic receptors or its signaling pathway. Therefore, we tested whether DHS could mimic other aspects of catecholamine stimulation, such as adrenergic driven gene expression.

### 3.2. DHS enhances isoproterenol-dependent gene expression

**Fig 3:**
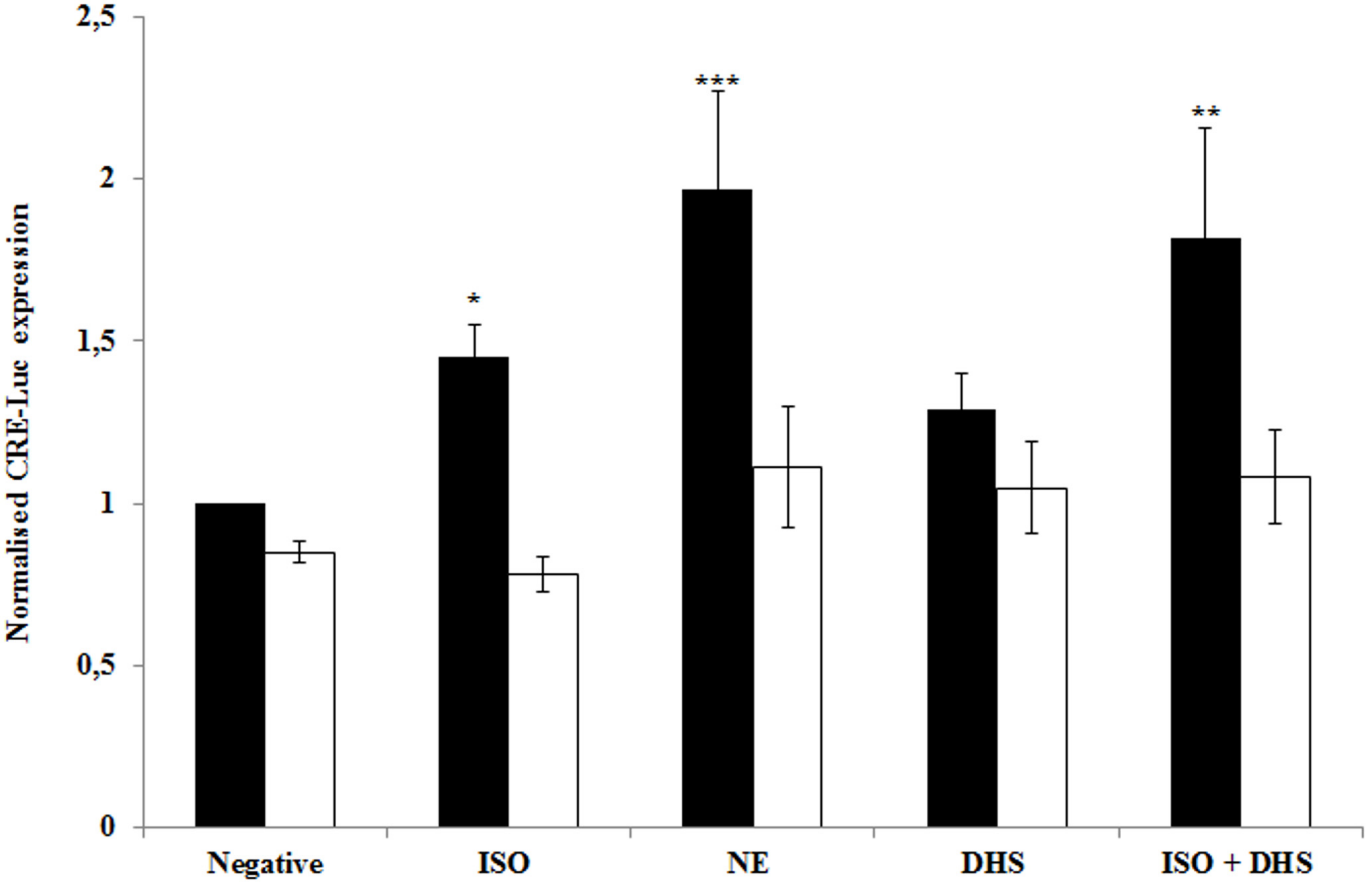
Effect of DHS on cAMP-dependent gene expression. A chart of relative activity of CRE-driven luciferase in dual-transfected H9c2 cells, treated with either isoproterenol (ISO, 1 μM), norepinephrine (NE, 100 μM), 2,3-dehydrosilybin (DHS, 25 μM) or a combination of ISO and DHS. A parallel group was treated with the aforementioned compounds and propranolol (PRO, 5 μM). The chart shows a significant increase in relative luciferase activity in cells treated with NE or co-treated with ISO and DHS. β-galactosidase activity served as the baseline for calculating the fold induction. Data are average ± SD for three independent experiments, each experiment was performed as triplicates. Values are significantly different from control values * (p < 0.05); ** (p < 0.01); *** (p < 0.001).

Adrenergic driven gene expression may be tested in transfected model cell line H9c2 which expresses both β1- and β2-adrenergic receptors [19]. It was found that 24 h post transfection DHS at concentrations up to 50 μM did not significantly increase relative cyclic AMP-responsive element (CRE)-driven luciferase activity in our H9c2 model, unlike 100 μM NE, which increased this expression twofold (P<0.001) (Fig 3, S1 Fig). We also tested NE at lower concentrations, 10 and 1 μM, for which we obtained similarly increased luciferase expression. Curiously, when cells were co-treated with ISO and DHS, there was a significant increase in CRE driven luciferase expression. 5 μM propranolol (PRO), which we used as a control inhibitor/antagonist, prevented this increase in luciferase expression. These results indicate that DHS sensitises cells to adrenergic stimulation, potentially through a cAMP responsive mechanism. Since activation of cAMP responsive mechanisms may be accompanied by an inhibition of phosphodiesterases and is marked by a rise in cellular cAMP concentration, we proceeded to test for an increase in cAMP concentration and phosphodiesterase inhibition.

### 3.3. Effect of DHS on phosphodiesterase activity and cAMP accumulation

**Fig 4:**
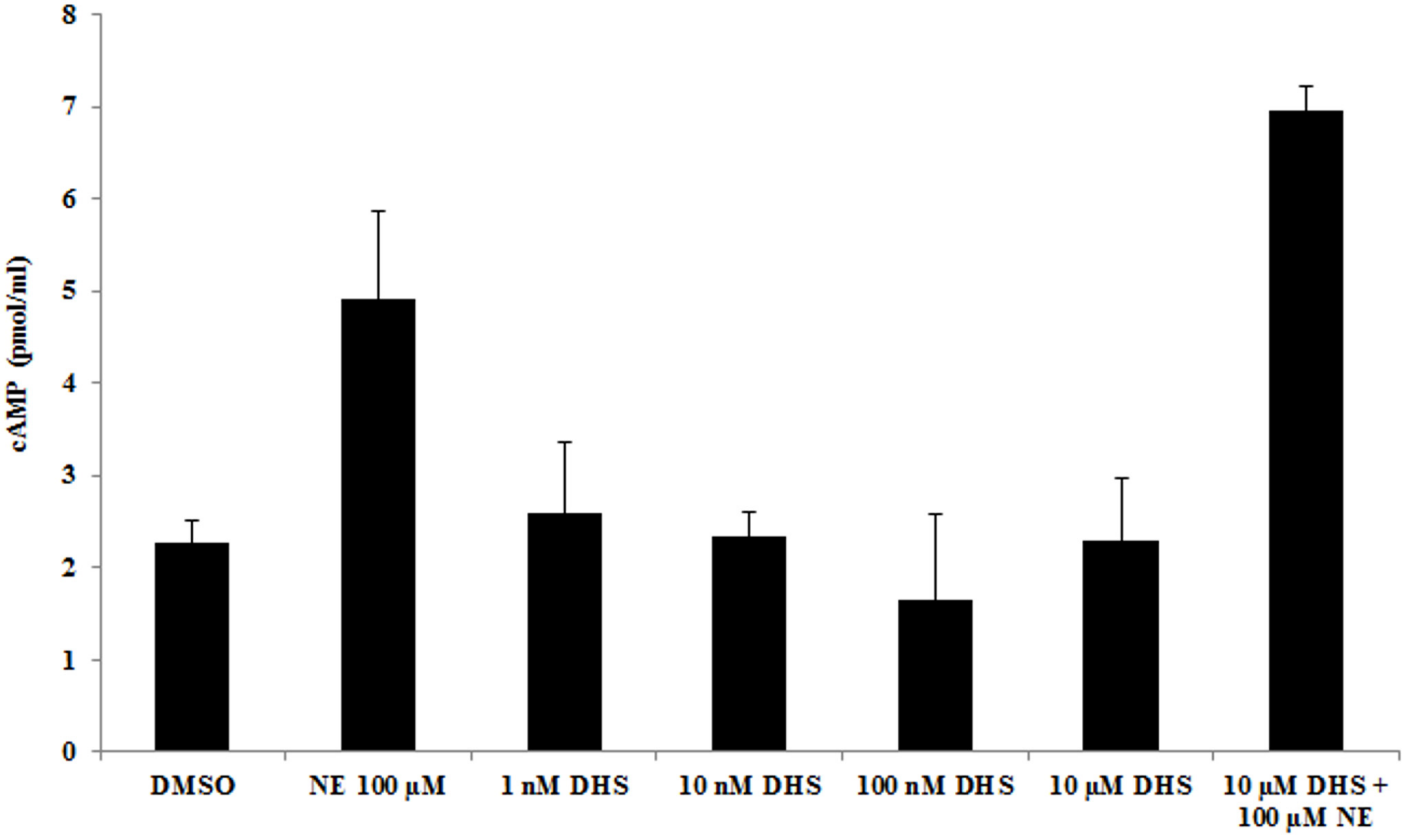
Effect of DHS on phosphodiesterase activity. Phosphodiesterase activity was evaluated in the presence of various DHS concentrations or in the presence of 50 μM IBMX. Data are average ± SD for three independent experiments, each experiment was performed as triplicates. Values are significantly different from control values * (p < 0.05).

Phosphodiesterase (PDE) inhibition assay revealed that ≥10 μM DHS concentrations inhibit PDE activity (Fig 4). However, even the highest DHS concentration tested was unable to reach the inhibitory effect of 50 μM IBMX, a known non-specific PDE inhibitor, which was used for comparison. Increasing DHS concentration above 10 μM did not result in further increase of the inhibitory effect, perhaps due to solubility limitations. Similar solubility limitations related to DHS have been noted by others [8]. Hence, inhibition was not dose dependent, with an abrupt increase in inhibition of PDE activity at 10 μM DHS.

**Fig 5:**
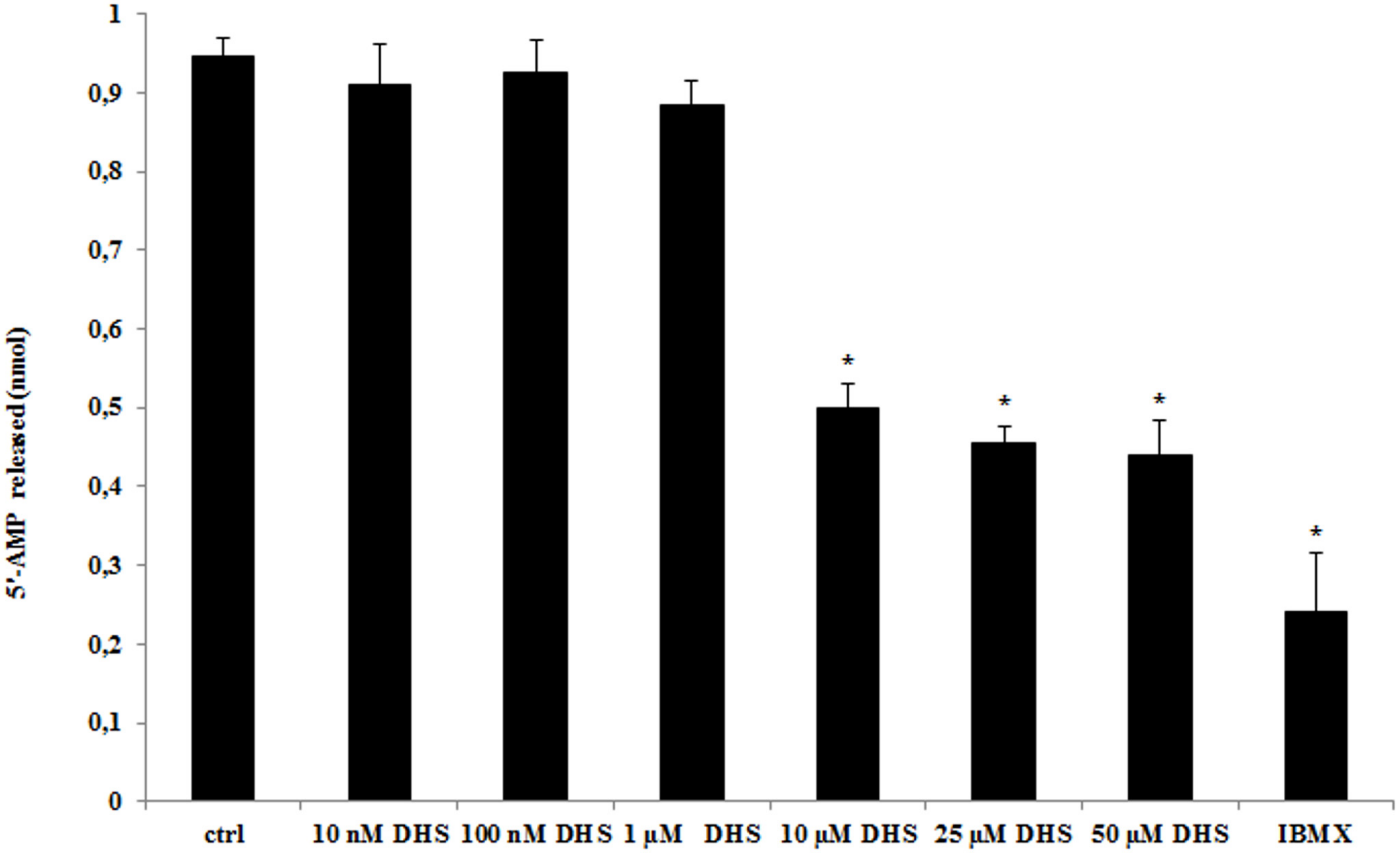
Effect of DHS on cAMP accumulation. Isolated neonatal rat cardiomyocytes were subjected to treatments with DMSO (control), NE or various concentrations of DHS alone or in combination for 5 min followed by measurement of intracellular cAMP level using ELISA based assay. Data are average ± SD for three independent experiments. Values are significantly different from control values * (p < 0.05).

Moreover, DHS failed to trigger significant cAMP accumulation in isolated neonatal rat cardiomyocytes at all concentrations tested (Fig 5). 100 μM NE caused significant increase in intracellular cAMP following 5 min of incubation thereby verifying the involvement of adrenergic pathway. The effect of NE was not diminished in the presence of DHS. On the contrary, DHS appeared to somewhat enhance the effect of NE but the increase did not reach statistical significance.

Our experiments with transfected H9c2 cells, neonatal cardiomyocytes and phosphodiesterase did not support the role of DHS as a potential adrenergic agonist. Therefore we set out to test whether prior depletion of catecholamines would influence the effect of DHS on whole hearts.

### 3.4. Reserpine treatment diminishes the positive inotropic effect caused by DHS

Reserpine is an indole alkaloid that was used for hypertension treatment as early as the 1940s. It inhibits the vesicular monoamine transporter, thereby preventing norepinephrine and dopamine uptake into storage vesicles and hence enhancing the degradation of released catecholamines by cytoplasmic monoamine oxidase [20]. The result is depletion of catecholamines from the sympathetic nerve endings. Therefore we tested the influence of reserpine pre-treatment, i.e. depletion of catecholamines, on DHS inotropic effect.

**Fig 6:**
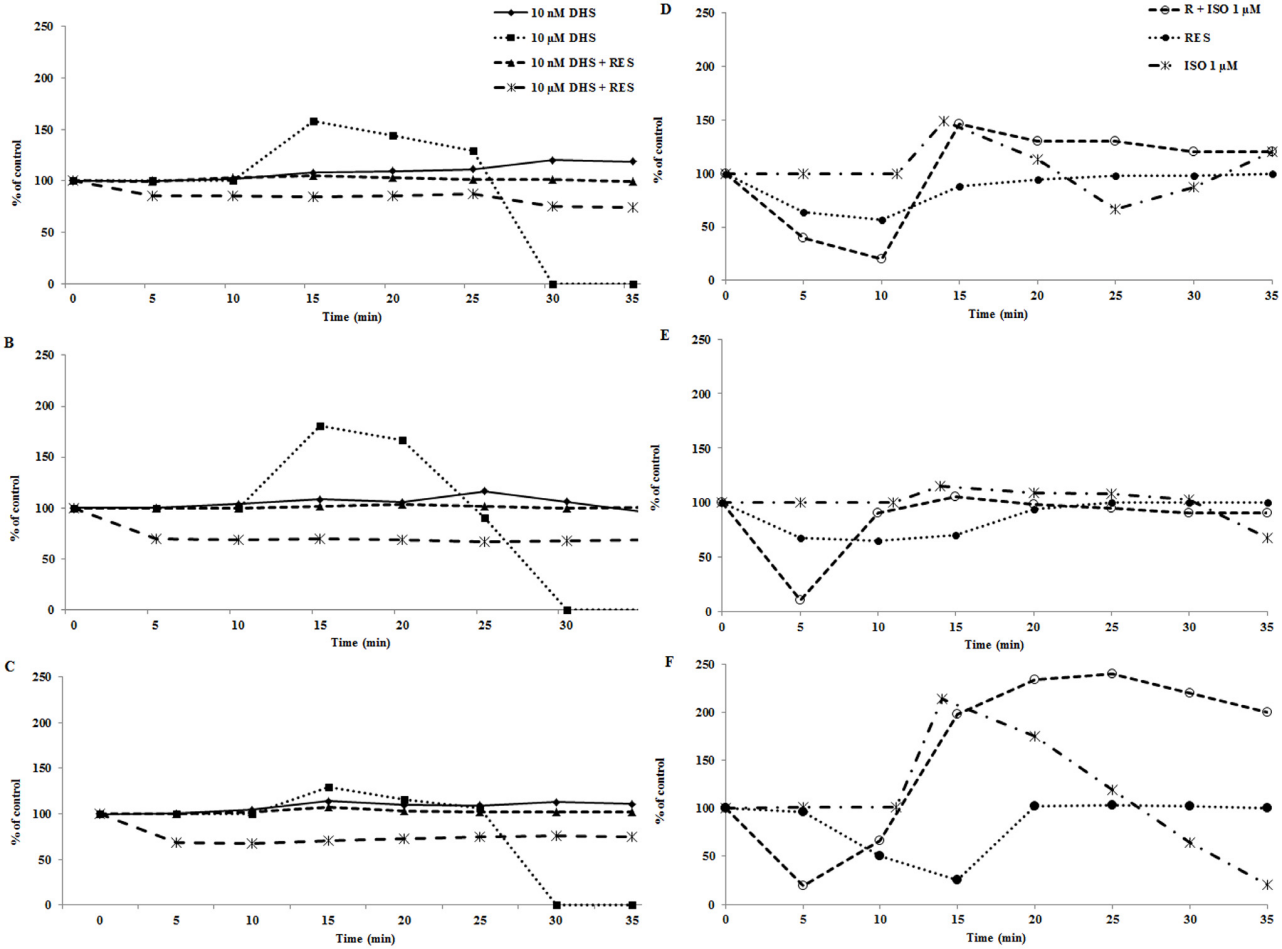
Effect of reserpine pre-treatment on the DHS activity in perfused hearts. Adult rat hearts were isolated from animals given 0.3 mg reserpine per kg of body weight 24 h prior to the surgical procedure. The hearts were perfused with Krebs-Henseleit buffer for 10 min followed by 15 min perfusion with DHS or Krebs-Henseleit buffer in control hearts, and concluded by 10 min perfusion (wash-out) with Krebs-Henseleit buffer. Data presented were obtained for hearts treated with: 10 nM DHS (◆, full line); 10 μM DHS (■, dotted line); 10 nM DHS + reserpine (▲, dashed line) and 10 μM DHS + reserpine (*, dashed line). Individual panels are: A—LVP; B—contractility; C—heart rate. Each line represents an average for at least four experiments. Data presented were obtained for hearts treated with: reserpine (●, dotted line); 1 μM ISO (*, dash-dot-dash line); and 1 μM ISO + reserpine (○, dashed line). Individual panels are: D—LVP; E—contractility; F—heart rate.

We observed signs of lethargy and docility in rats treated with reserpine for 24 h prior to experiment, which is in keeping with the known effects of reserpine. Whole hearts isolated from these animals displayed lower LVP and slight bradycardia as compared to hearts excised from control animals not treated with reserpine. When DHS was applied to the perfused hearts isolated form reserpine-treated rats, the inotropic effect of DHS was abolished (Fig 6). In fact there was no increase in LVP, contractility or heart rate during the 10 μM DHS perfusion. The hearts remained stable, with parameters displaying no significant difference from control hearts. A similar lack of inotropic effect was seen with 10 nM DHS, with hearts maintaining normal biomechanical parameters throughout the perfusion period. On the other hand, reserpinised heart perfused with 1 μM ISO displayed a rapid response similar to that seen in control heart. Therefore stimulation of β-adrenergic receptors was maintained in the reserpine treated hearts. It must be noted though, that hearts excised from reserpine pre-treated animals and not treated with DHS displayed initial loss of activity, i.e. loss of LVP, contractility and heart rate, followed by recovery of biomechanical parameters at 10 min of perfusion. We can speculate that this initial lag phase is due to the depletion of catecholamines. In one isolated case, which is not included in the data presented, the heart did not recover and reached cardiac arrest after 25 min of perfusion.

## Discussion

Polyphenolic compounds are generally viewed as dietary antioxidants which protect biological systems from oxidative damage by modulating cellular reactive oxygen species (ROS) levels by virtue of their radical-scavenging activities, either as individual substances or as a complex mixture [21]. Naturally, the antioxidant properties were, and still are, a major research focus. However, more recent lines of evidence suggest that the biological activities of flavonoids, and their corresponding *in vivo* metabolites, depend on the modulation of specific protein functions, including intracellular cell signaling and receptor activities [22–24].

Myocardium is strictly aerobic and hence vulnerable to a decrease in oxygen supply with dire, potentially fatal, consequences. The extent of damage arising from pharmacological treatments and/or ischemia/reperfusion is highly dependent on intracellular signaling and modulation thereof by various means, including dietary and pharmacological agents. The complexity of the signaling involved in cardioprotection (cf. [13]) offers numerous venues for exploring the cardioprotective activity of polyphenols (cf. [25]).

Silymarin and a few of its constituents were tested for and demonstrated to display cardioprotective activities. We have previously showed that DHS reduces free radical production and de-energises mitochondria [11]. As depolarisation of the mitochondrial membrane leads to an increase in cytoplasmic calcium concentration [26] and lowered ATP levels [27], it may also lead to a depolarisation of the plasma membrane. We would postulate that the effects of DHS that we have observed may be due to these effects of cellular de-energisation. This would lead to two effects which are relevant to the observations made in this study. One of the effects of depolarisation of the plasma membrane includes the release of neurotransmitter from presynaptic vesicles. When this happens in sympathetic terminals in the heart, the release of catecholamines would have inotropic effects similar to those observed in this study. In addition, as catecholamines have been found to be capable of preconditioning and post conditioning the myocardium against ischemia/reperfusion injury [28], the catecholamine-like activity of DHS shown here is consistent with the cardioprotective effect of DHS seen by Gabrielova *et al*. [12]. Secondly, the increase of cytoplasmic Ca^2+^ in and of itself would activate CamKII, which would then act on PLCγ and the IP3K/Akt cascade. This also leads to pre- or post- conditioning via activation of PKCε, essentially utilising the same intracellular pathways as the adrenergic pathway. In addition, an increase in cytoplasmic calcium would act directly to increase contractility of cardiomyocytes.

The protective effects of ischemic postconditioning in the isolated heart model were shown to depend on PKCε [29,30]. PKCε is activated by a series of phosphorylations in its catalytic domain, with phosphorylation at Ser729 cardioprotection increases [31] and loss of the Ser729 phosphate is associated with PKCε translocation to the cell periphery or nucleus [32]. Our previous results have shown a significant decrease in the Ser729–phosphorylated pPKCε relative to the total PKCε following hypoxia-reoxygenation of cardiomyocytes, which is prevented by post-conditioning of the cells with DHS [12]. Hence DHS likely affects signaling pathways, including adrenergic, that influence PKCε phosphorylation status.

Our suggestion of possible cardioprotective effect of DHS is based on reports by Brennan et al. [33,34] who have shown that carbonyl cyanide 4-(trifluoromethoxy)phenylhydrazone (FCCP), an artificial uncoupler of oxidative phosphorylation, is cardioprotective at low concentrations while higher concentrations of the substance are detrimental to the heart. In our view, this is a more general phenomenon. For example reactive oxygen species serve as signaling molecules at low concentrations while causing damage at high concentrations. Therefore, low concentrations of DHS that are not causing arrhythmias could be cardioprotective. Cardioprotection may stem from modulating ROS signaling and mitochondrial function, as shown in our previous publications [11,12], or from beta-preconditioning, as catecholamine-like effect of DHS presented here.

Data presented here clearly show that DHS exhibits an inotropic effect on excised rat hearts and that this effect of DHS is absent in animals pre-treated with reserpine, *i*.*e*. that the inotropic effect at all concentrations is dependent on the presence of catecholamines. Furthermore, treatment of reserpinised hearts with ISO demonstrates that even following depletion of endogenous catecholamines, exogenous β-agonists, unlike DHS, still have an inotropic effect. In addition, the lack of effect on the intracellular concentration of cAMP, despite the inhibition of PDE by high concentrations of DHS, undermines the apparent β-agonist activity of DHS. Moreover, DHS alone was incapable of eliciting catecholamine-dependent gene expression in our model cell line. Finally, ISO, unlike DHS, evoked an inotropic effect in both reserpinised and nonreserpinised hearts, demonstrating the independence of β-agonist stimulated inotropy from endogenous catecholamines.

There are several implications of the findings presented here. The first and foremost is that DHS at low nanomolar concentration triggers positive inotropic effect. It suggests that silymarin, which contains trace amounts of DHS, may exert its cardioprotective activity through stimulation of adrenergic pathway. However, the effect of low nanomolar DHS is not profound enough to be noticeable in *in vivo* experiments, i.e. the animals are unlikely to develop sustained tachycardia or hypertension due to administration of silymarin.

By the same logic, a sufficiently high dose of purified DHS, administered intravenously would likely exhibit inotropic effects *in vivo*, as it does in perfused hearts. Furthermore, as the release of catecholamines is a key step in the mechanism of ischemic preconditioning [35], and our results suggest that the inotropic effects of DHS are likewise due to the release of catecholamines, there is a strong case for *in vivo* studies into pre- and post-conditioning by DHS.

The structure of DHS is also noteworthy. While it does not resemble any known substances currently used for treating cardiovascular problems, DHS forms an interesting parent compound to be explored. A recent study examining the effects of galloyl derivatives of silybin on angiogenesis, found that the flavonolignan and most of its derivatives lacked anti-angiogenic activity [36]. Extrapolating these results to DHS, we could speculate that the same will be true. While this is disheartening for oncologists, it should encourage the exploration of the use of DHS for the treatment of cardiovascular disorders.

Lastly, the lack of apparent β-agonist or β-antagonist activity of DHS lowers the probability of negative side effects if silymarin, or DHS alone, are combined with other cardiovascular treatments.

## Conclusion

Taken together, our data demonstrate DHS as a novel inotropic agent whose activity relies on the presence of endogenous catecholamines. DHS, with its previously shown influence on PKCε, may prove to be a positive modulator of cardiac functions under various (patho)physiological conditions.

## Supporting Information

S1 Fig Effect of DHS on cAMP-dependent gene expression. A chart of relative activity of CRE-driven luciferase in dual-transfected H9c2 cells, treated with either norepinephrine (NE, 100 μM), or various concentrations of 2,3-dehydrosilybin (DHS). A parallel group was treated with the aforementioned compounds and propranolol (PRO, 5 μM). The chart shows a significant increase in relative luciferase activity in cells treated with NE, there is no statistically significant change in any of the DHS concentrations tested. β-galactosidase activity served as the baseline for calculating the fold induction. Data are average ± SD for three independent experiments, each experiment was performed as triplicates.
(TIF)
